# Alpha-1-proteinase inhibitor regulates CD4 lymphocyte levels and is rate limiting in HIV-1 disease

**DOI:** 10.1186/1742-4690-9-S1-P23

**Published:** 2012-05-25

**Authors:** Cynthia L Bristow, Mariya A Babayeva, Michelle Labrunda, Michael P Mullen, Jose Cortes, Ronald Winston

**Affiliations:** 1 Weill Cornell Medical College, New York, USA

## Introduction

Adult stem cell migration through human hematopoietic tissue requires the chemokine CXCL12 and its receptor CXCR4. In addition, human leukocyte elastase (HLE) plays a key role. When HLE is located on the cell surface (HLECS), it acts not as a proteinase, but as a receptor for α1proteinase inhibitor (α_1_PI, α1antitrypsin). Binding of α_1_PI to HLECS forms a motogenic complex. We previously demonstrated that α_1_PI deficiency attends HIV-1 disease. Here we investigate the mechanism and therapeutically address the α_1_PI deficiency of HIV-1 infection.

## Materials and methods

Blood was collected from 30 HIV-1 uninfected and 39 HIV-1 infected adults. Residual sera was obtained from 20 HIV-1 uninfected chimpanzees, 2 chimpanzees pre- and 42 months post-HIV-1 challenge, 12 HIV-1-immunized macaques, and 3 SHIV-infected macaques. Three HIV-1 infected individuals received α_1_PI augmentation therapy.

## Results

In HIV-1 uninfected individuals, CD4^+^ lymphocytes were correlated with the combined factors α_1_PI, HLECS+ lymphocytes, and CXCR4+ lymphocytes (r^2^ = 0.91, p < 0.001, n = 30), but not CXCL12. In contrast, in HIV-1 individuals with >220 CD4 cells/µl, CD4+ lymphocytes were correlated solely with active α1PI (r^2^ = 0.93, p < 0.0001, n = 26). The monoclonal anti-HIV-1 antibody 3F5 present in HIV-1 patient blood bound and inactivated human α_1_PI. Chimpanzee α_1_PI differs from human α_1_PI by a single amino acid which lies within the 3F5-binding epitope. Unlike human α_1_PI, neither chimpanzee nor macaque α_1_PI bound to 3F5, nor was α_1_PI depleted following HIV-1 challenge, consistent with the normal CD4^+^ lymphocyte numbers of HIV-1 infected chimpanzees. The presence of IgG- α_1_PI immune complexes correlated with decreased CD4^+^ lymphocytes in HIV-1 individuals, and α1PI augmentation quadrupled the number of immunocompetent CD4^+^ lymphocytes with no untoward effects.

## Conclusions

An autoimmune component of HIV-1 disease was identified and was overcome therapeutically. Results identify an achievable vaccine modification with the novel objective to protect against AIDS as opposed to the current objective to protect against HIV-1 infection.

